# Supplementary material for: Radiomics-Based Machine Learning Model for Diagnosis of Acute Pancreatitis Using Computed Tomography
Source: Diagnostics (Basel). 2024 Mar 28;14(7):718. doi: 10.3390/diagnostics14070718 (PMC11011980; doi:10.3390/diagnostics14070718)
Supplement: Supplementary file 1 [file diagnostics-14-00718-s001.zip › diagnostics-2880562-supplementary.pdf]

Supplemental Table S1 Importance of all analyzed features in a random forest based model

	Mean	Median	Minimum	Maximum	Decision
shape_Elongation	0.20963917	0.48971637	-2.29549088	1.5923146	Rejected
shape_Flatness	2.42320248	2.44573467	-0.37194873	4.2236132	Rejected
shape_LeastAxisLength	5.78388200	5.86566265	4.41601723	7.2530128	Confirmed
shape_MajorAxisLength	1.43935261	1.46289603	-0.25099999	3.3659761	Rejected
shape_Maximum2DDiameterColumn	-0.09916046	-0.14336380	-2.03357077	1.5117521	Rejected
shape_Maximum2DDiameterRow	0.38906406	0.58001607	-1.90212829	2.4105838	Rejected
shape_Maximum2DDiameterSlice	0.66038835	0.90508725	-0.81539552	2.0052965	Rejected
shape_Maximum3DDiameter	4.58723576	4.65645036	1.87389796	6.3156002	Confirmed
shape_MeshVolume	9.23885788	9.25235686	7.46715843	10.2188769	Confirmed
shape_MinorAxisLength	-0.54527222	-0.64149706	-1.78315801	0.7924367	Rejected
shape_Sphericity	5.00638885	4.97530508	3.49440801	7.1465528	Confirmed
shape_SurfaceArea	5.76865803	5.70833370	4.25346085	7.0809316	Confirmed
shape_SurfaceVolumeRatio	11.66042443	11.70966218	9.09119950	13.8427469	Confirmed
shape_VoxelVolume	9.11541488	9.12006238	7.51713260	10.3528376	Confirmed
firstorder_10Percentile	3.51198151	3.58138335	1.29672870	5.3842298	Tentative
firstorder_90Percentile	1.35067410	1.25248299	0.18361485	2.6577618	Rejected
firstorder_Energy	9.01044035	8.94489517	7.87366862	10.5241848	Confirmed
firstorder_Entropy	0.81872418	0.87466340	-1.18045396	2.5434038	Rejected
firstorder_InterquartileRange	4.05458399	4.05878301	1.64074558	6.1597799	Confirmed
firstorder_Kurtosis	4.25903797	4.30577918	1.85875113	6.1540773	Confirmed
firstorder_Maximum	5.37865681	5.39767457	2.57683703	8.0169613	Confirmed
firstorder_MeanAbsoluteDeviation	1.26727323	1.48001861	-1.06576337	2.7572792	Rejected
firstorder_Mean	1.80831253	1.93518989	0.66874905	3.2224625	Rejected
firstorder_Median	1.84965293	2.12439544	0.34469308	2.8202230	Rejected
firstorder_Minimum	5.25882003	5.32104944	3.47374084	7.4919898	Confirmed
firstorder_Range	1.72615114	1.73171447	0.50600479	2.6698976	Rejected
firstorder_RobustMeanAbsoluteDeviation	4.03306478	4.05641341	1.80130759	6.4914035	Confirmed
firstorder_RootMeanSquared	1.79498591	1.81243315	0.55121552	3.2675896	Rejected
firstorder_Skewness	7.27757582	7.40393305	4.68083471	10.0629144	Confirmed
firstorder_TotalEnergy	8.91737564	8.94917816	7.15839578	9.9716422	Confirmed

firstorder_Uniformity	1.76191944	1.90824417	1.08275008	2.2311903	Rejected
firstorder_Variance	1.07937780	1.38134492	-0.86725575	1.8599638	Rejected
glcm_Autocorrelation	2.96217649	2.99927513	0.60674719	4.9166821	Tentative
glcm_JointAverage	2.96096394	2.90170353	0.92667826	4.6206019	Tentative
glcm_ClusterProminence	1.85263748	1.86655841	0.15854996	3.3951387	Rejected
glcm_ClusterShade	4.07142517	3.95468526	1.99449083	6.0568867	Confirmed
glcm_ClusterTendency	0.95001811	0.70179011	-0.82326599	3.6225971	Rejected
glcm_Contrast	1.06022214	1.33921096	-1.15744551	2.4823831	Rejected
glcm_Correlation	0.58589491	0.62369369	-1.41944943	2.2310027	Rejected
glcm_DifferenceAverage	1.04474279	1.11496337	-0.51654316	2.1917428	Rejected
glcm_DifferenceEntropy	0.34820559	0.48630027	-0.84419707	2.2817829	Rejected
glcm_DifferenceVariance	1.03802976	1.14618546	-0.97158927	2.8510740	Rejected
glcm_JointEnergy	2.83673134	2.86303216	0.30157652	5.0954904	Tentative
glcm_JointEntropy	0.43600548	0.62400558	-1.20595127	2.2571025	Rejected
glcm_Imc1	0.96719022	0.73225517	-0.35281686	2.6105018	Rejected
glcm_Imc2	1.27638772	1.45950138	-0.38929169	2.5850083	Rejected
glcm_Idm	0.90686025	1.02154512	-1.23703896	2.6871991	Rejected
glcm_Idmn	2.13410138	2.24944718	-0.32386031	3.6736523	Rejected
glcm_Id	0.89423747	1.16457593	-0.62462371	2.0361505	Rejected
glcm_Idn	2.31487653	2.34819823	0.91962655	3.7052605	Rejected
glcm_InverseVariance	1.65284681	1.55516764	0.15691831	3.1975394	Rejected
glcm_MaximumProbability	2.79614785	2.85536844	0.54216590	5.2519959	Tentative
glcm_SumEntropy	1.54305000	1.81867070	-1.13832553	3.2983994	Rejected
glcm_SumSquares	0.47286578	0.68748078	-1.01858370	2.2082297	Rejected
glrlm_GrayLevelNonUniformity	5.85097717	5.87887549	3.63440264	7.1486559	Confirmed
glrlm_GrayLevelNonUniformityNormalized	1.50291385	1.54170968	0.21471887	3.1349701	Rejected
glrlm_GrayLevelVariance	1.17771379	1.30765278	-0.68378357	2.1155719	Rejected
glrlm_HighGrayLevelRunEmphasis	2.49782178	2.54543927	1.29986454	3.2418100	Rejected
glrlm_LongRunEmphasis	0.54373435	0.03024667	-0.42742231	1.8983498	Rejected
glrlm_LongRunHighGrayLevelEmphasis	3.05238602	3.01574621	0.63490525	5.2366467	Tentative
glrlm_LongRunLowGrayLevelEmphasis	2.30045611	2.35253741	0.22356168	3.7723488	Rejected
glrlm_LowGrayLevelRunEmphasis	1.54478106	1.59884578	0.33350204	2.5459692	Rejected

glrlm_RunEntropy	0.23633749	0.33360268	-1.04243702	1.7659448	Rejected
glrlm_RunLengthNonUniformity	7.76891287	7.78902528	6.37625470	9.2211188	Confirmed
glrlm_RunLengthNonUniformityNormalized	1.51715095	1.50543697	0.54438576	2.6051579	Rejected
glrlm_RunPercentage	1.65365727	1.95571214	-0.86681467	2.9758579	Rejected
glrlm_RunVariance	1.16973319	1.16944483	-0.32906954	2.7001232	Rejected
glrlm_ShortRunEmphasis	1.86208395	2.07216419	0.40041362	3.4153822	Rejected
glrlm_ShortRunHighGrayLevelEmphasis	1.96609333	1.84006944	1.27922256	3.2679194	Rejected
glrlm_ShortRunLowGrayLevelEmphasis	1.96440500	2.13732033	0.84065458	2.9978123	Rejected
glszm_GrayLevelNonUniformity	3.98142304	4.07691651	1.89831000	5.6817014	Confirmed
glszm_GrayLevelNonUniformityNormalized	0.97836107	1.33489219	-1.31686299	2.1076497	Rejected
glszm_GrayLevelVariance	1.54025875	1.39920466	0.46254909	2.9451254	Rejected
glszm_HighGrayLevelZoneEmphasis	1.29978128	1.48395131	-0.45537443	2.3431475	Rejected
glszm_LargeAreaEmphasis	4.45249991	4.42380815	3.10441514	5.8229960	Confirmed
glszm_LargeAreaHighGrayLevelEmphasis	1.49024134	1.56077412	-0.55233625	2.9330464	Rejected
glszm_LargeAreaLowGrayLevelEmphasis	4.99167260	5.00927317	3.09797560	6.4373932	Confirmed
glszm_LowGrayLevelZoneEmphasis	1.20395872	1.28459154	-0.55066436	2.4458819	Rejected
glszm_SizeZoneNonUniformity	3.05996695	3.05848646	1.07699583	4.8852722	Tentative
glszm_SizeZoneNonUniformityNormalized	0.83917414	1.02912731	-0.56963038	2.3597478	Rejected
glszm_SmallAreaEmphasis	1.19263693	1.48036744	-0.22311150	2.1733612	Rejected
glszm_SmallAreaHighGrayLevelEmphasis	1.28817495	1.22014317	-1.36203263	2.8929392	Rejected
glszm_SmallAreaLowGrayLevelEmphasis	1.13888586	0.87291937	-0.38718983	3.0949663	Rejected
glszm_ZoneEntropy	1.33471078	1.58867723	-0.21555068	2.4468728	Rejected
glszm_ZonePercentage	5.00162175	5.00882786	2.98730446	6.7334022	Confirmed
glszm_ZoneVariance	4.41800856	4.51563720	2.69168804	5.9308521	Confirmed
gldm_DependenceEntropy	1.18161994	1.56159913	-1.13460341	2.8745154	Rejected
gldm_DependenceNonUniformity	10.96221136	11.13097391	8.54542612	12.8445948	Confirmed
gldm_DependenceNonUniformityNormalized	4.51733098	4.48688664	2.34459607	6.3799840	Confirmed
gldm_DependenceVariance	4.96678640	5.01815975	2.60574703	7.1888311	Confirmed
gldm_GrayLevelNonUniformity	3.89292678	3.88923839	2.16768636	5.4180304	Confirmed
gldm_GrayLevelVariance	0.66176709	0.78815226	-1.14311790	2.0240843	Rejected
gldm_HighGrayLevelEmphasis	2.49737051	2.60838586	0.07231512	3.9395173	Rejected
gldm_LargeDependenceEmphasis	1.04361487	1.09120050	-0.56283247	2.7247942	Rejected

gldm_LargeDependenceHighGrayLevelEmphasis	3.03332742	3.04978462	1.02329084	4.6856391	Tentative
gldm_LargeDependenceLowGrayLevelEmphasis	2.28031351	2.45878947	0.27000664	3.9242001	Rejected
gldm_LowGrayLevelEmphasis	2.01800600	1.93397330	0.75369701	3.8278209	Rejected
gldm_SmallDependenceEmphasis	2.92368800	2.93723149	1.64098471	4.2567670	Tentative
gldm_SmallDependenceHighGrayLevelEmphasis	1.68455654	1.80430983	0.08859649	2.6622981	Rejected
gldm_SmallDependenceLowGrayLevelEmphasis	1.41928524	1.68315308	-0.61893119	2.5185854	Rejected
ngtdm_Busyness	2.42258370	2.39936564	0.31966934	4.0439238	Rejected
ngtdm_Coarseness	5.24710430	5.25194846	3.33952740	6.5647794	Confirmed
ngtdm_Complexity	1.33039668	1.42345122	-0.11354229	3.0676271	Rejected
ngtdm_Contrast	0.81413695	1.05621058	-0.95910710	2.3720056	Rejected
ngtdm_Strength	2.99753322	2.97033813	0.72773784	4.8129675	Tentative
